# Progress in Surgery

**Published:** 1896-04-18

**Authors:** 


### Progress in Surgery.

### GENERAL SURGERY.

**Antiseptics.**—In "A Brief Note on Aseptic Surgery," C. B. Lockwood[1] alludes to recent experiments which tend to shake the position of some of our favourite chemicals. An objection which greatly detracts from the germicidal properties of corrosive sublimate and carbolic acid is that they combine with albumen to form compounds which are quite inert; while biniodide of mercury has no such effect. These experiments show that staphylococcus pyogenes aureus, staphylococcus pyogenes, and the other common pyogenic cocci possess great powers of resistance against sublimate and carbolic acid. A 1 in 1,000 solution of sublimate killed aureus in two minutes; and carbolic lotion, 1 in 100, took from 15 to 17 minutes. This exemplifies their action under favourable circumstances, and without the presence of blood or pus. Lockwood's own attempts at the disinfection of the human skin, septic ulcers, or sinuses gave exactly similar results; indeed, he has become sceptical whether it is possible with chemicals to disinfect an ulcer or sinus. Hænel's researches[2] tend to prove the same. He believes that the best means of producing recovery is to provide free drainage for the secretions of the wound, to keep the wound open, and absorb all the osmotic secretions in the dressing. Lockwood insists upon the absolute necessity of surgeons using tests to the skin during operations.

In a most exhaustive review of the subject, W. Poliakoff[3] considers the important question, whether an essential difference exists between bacterial and non-bacterial suppuration. He concludes that the characteristics usually offered of bacterial suppuration are erroneous, and occur in the non-bacterial form, viz., that (1) it manifests a disposition to progress; (2) the pus consists of multinucleated pus corpuscles; (3) the pus contains peptone. It is probable that constant and long-continued excretion of toxins into the tissues, as occurs in nature, produces the reaction of the tissues which is known as suppuration. The cause of stitch abscesses observed in patients after aseptic operations has been discussed by Lauenstein[4] before the German Surgical Congress. His 216 observations on different sutures and ligatures tend to confirm the suspicion that catgut is a frequent source of infection in wounds. Since 1888 Kocher has used silk only in his goitre operations, and has had 85 per cent. absolute primary healings.

P. Reclus' manner of treating extensive crushing injuries to the limbs by conservative methods has been recently described before the French Congress of Surgery.[5] Whatever the extent or gravity of the lesions, he never, under any circumstances, amputates the injured limb, but merely wraps it in antiseptic substances by a veritable "embalming" process, leaving nature to separate the dead from the living tissues. This method of treatment possesses the double advantage of being much less fatal than surgical exæresis, and of preserving for the use of the patient, if not the entire limb, at any rate a much larger part than would be left after amputation. He advocates this very conservative treatment on account of the excellent effects of hot water, which he uses freely. After the skin has been shaved and cleansed from all fatty substances by ether, &c., in the usual way, a jet of hot water 60° to 62° C., but not higher, is made to irrigate all the injured surfaces, and to penetrate into all the hollows and under all the detached parts of the wound, without exception. This is the only way of removing all clots, and to wash away all foreign bodies, together with the micro-organisms they may contain. The advantages of hot water at this high temperature are threefold—(1) hot water at this temperature is antiseptic, heat greatly increases the potency of antiseptic substances; (2) is hæmostatic; (3) it helps to compensate for the loss of heat, resulting from the bleeding, and especially from the traumatic shock. After the "embalming" process, and the dead tissue has been separated from the living, the surgeon has nothing to do except to divide the bone at a suitable spot. According to Reclus,[6] the results obtained are remarkable.

With a view to find an agent of the greatest desiccating power, most energetic as an antiseptic, and at the same time inimical to the tissues, E. R. W. Frank[7] has studied the action of the various iodine preparations, iodoform, dermatol, europhen, aristol, and nosophen. The best results followed the use of nosophen. It is tetra-iodo-phenol-phtalein, a yellowish odourless crystal, soluble only in ether, non-toxic, of acid reaction, but combines with alkalies, being then freely soluble in water. It prevents the emigration of leucocytes, and thus, like iodoform, is an agent which retards pus formation. H. W. Mitchell[8] has given a rather full description of his extensive experience with a new antiseptic fluid, which he states is simply a solution of halogen salts chemically combined. Oxycyanide of mercury, first recommended by Chibret, has been used for the past four years by C. Monod and Magaigne.[9] These authors state that in 5 per 1,000 solution it displays in laboratory experiments an antiseptic potency always equal to, and often greater than, that of 1 in 1,000 sublimate solution. It has no disadvantages beyond those of corrosive sublimate, but has the special advantage of not affecting either the hands or instruments. It should not be used for washing out cavities. In atonic and indolent ulcers occurring in persons of the poorer classes, A. Shirman[10] has employed commercial kerosene, either pure or diluted, with alcohol (33 to 50 per cent.). He applies it with a camel's hair brush, or with a piece of gauze soaked in the solution. The burning sensation felt at first soon passes away, and healing is produced in a comparatively short space of time. Camphorated salol has, according to J. T. Bowen,[11] been employed by Elsenburg, especially in furuncles and carbuncles. It is prepared by moistening one part of camphor with a few drops of alcohol, rubbing this in a porcelain mortar with 1·4 part of salol until a transparent liquid is obtained. Airol, a compound containing bismuth, gallic acid, and iodine, is recommended by Veiel[12] as a substitute for iodoform. He believes that it is in many cases superior to iodoform, in that it is inodorous, non-poisonous, and non-irritant, while at the same time it greatly diminishes secretion. Chinosol, a product belonging to the chinoline compounds, has also lately been brought forward as a powerful antiseptic, being non-toxic, non-caustic, and readily soluble, while it acts as an efficient deodoriser, and does not coagulate albumen.

**Fractures and Dislocations.**—Owing to the advance recently made in surgery, and especially in the introduction of antisepsis, immediate amputation, Heydenreich[13] observes, is no longer resorted to, except in cases of severe crushing of the bones, extensive lesions of the soft parts, or wounds involving the arteries or nerves to such an extent as to seriously jeopardise the vitality of the limb. Acute septic symptoms, such as rapidly extending gangrene, may also furnish an indication for amputation. In the same strain, Mayo Robson[14] remarks that compound fractures afford a bright example of progress in treatment, for instead of amputating where the large joints are opened, or where the bones are extensively crushed and the soft parts lacerated, and instead of the majority of cases having to go through a tedious process of healing by granulation, possibly with the complication of abscess and secondary hæmorrhage, and after many weeks or months recovering with stiffened joints and impaired functions, at the present time, although joints may be opened and the bones severely smashed, so long as the circulation can be carried on amputation scarcely enters into the question, and the limb is put up, after purification of the wound, with every prospect that the case will pursue the ordinary course of recovery like a simple fracture. In Heydenreich's opinion, in simple fractures there is but rarely any occasion for operation. In simple, as well as compound fractures, splinters may exist, and possibly lead to necrosis. Such pieces even when completely detached and deprived of their periosteum may again unite with the bone. There should, therefore, in a case of compound fracture, be no undue haste in treating splinters as foreign bodies, and operation should not be undertaken except after mature deliberation. If, however, a sharp point of bone threatens to injure the neighbouring soft parts (blood-vessels, nerves, or skin), operation is not only justifiable, but imperative. In certain impacted fractures, such as Colles's fracture of the wrist, at any age, or impacted fracture of the cervix femoris in a healthy young or middle-aged man, union is certain if the case be left to nature; but Robson believes that any surgeon would feel dissatisfied with his work if he simply rested content with securing union without restitution. In the same way, in transverse fracture of the patella, bony union and complete restitution, which were once rare events, are now definitely aimed at either by extra- or intra- articular suture. Bardeleben[15] gives the result of his treatment of fifty-eight fractures during the past year by the use of bandages, permitting the patient to walk about in all cases in which the condition did not demand an amputation, and in which the extremes of age did not make it dangerous. Joints that were not directly involved could be easily moved after the bandages were removed. Muscular atrophy, delirium, and senile catarrh were never present. The time required for healing was shorter than usual. Joseph Bell, Laing, Alexis Thomson, and others,[16] at the Medico-Chirurgical Society of Edinburgh, supported A. G. Miller in the treatment of fracture near a joint by rest, aided by massage and passive movement. Berger[17] thinks that no surgeon is justified in exposing the seat of fracture in the leg with the view of placing the fragments in good position, and of uniting them by wire, except in the very exceptional instances of persistent irreducibility due to the interposition of isolated pieces of bone and of bands of ligament, periosteum, or muscle. It is thought that only articular fracture of the elbow and knee, on account of the bad functional results so frequently observed after such injuries, should be subjected to primary surgical intervention. It is unjustifiable in simple fractures of the clavicle (the other long bone in which primary operation has been seriously proposed), except when this injury is complicated by vascular or nerve lesions. Langton[18] related an interesting case (in a patient aged 23) of osteopsathyrosis (Fragilitas Ossium) in which, after firm union of several fractures had taken place, disunion in two occurred several years afterwards. The left femur and right humerus became disunited, and the latter eventually became the seat of a sarcomatous growth. Disunion occurred many years after sound repair had taken place, and the patient was able to walk many miles in comfort on the ununited fracture. Wheelton Hind[19] divides ununited fractures as due to two classes of causes: 1. Systemic causes—syphilis, scurvy, wasting disease, insufficient food. 2. Mechanical causes—(1) non-apposition of fragments, which may be subdivided into separation of ends, as in fractured patella, and oblique fracture and misplacement of fragments, so that the raw and bony surfaces are not in contact; (2) the interposition of muscle or fascia between the ends of the bone; (3) necrosis of the ends of the bone in compound fracture; (4) rupture of the nutrient artery, the fracture being near the point of entrance; (5) imperfect blood supply to the limb owing to concomitant damage to the main vessels; (6) mobility of the fragments. Local treatment has for its object—first, rest and the proper apposition of the ends of the fragments; second, that the ends should be rendered immobile; third, that callus formation should be excited. To this end tenotomy, careful splinting, rubbing the ends of the bones together, counter irritation by blistering, friction, or seton. Failing these, subcutaneous incision, driving ivory pegs into the ends, simple resection, resection with pegging, ligature, or screwing, and finally bone grafting after resection. The method of treating dislocations, sprains, and contusions by massage grows in popularity, as attested by the frequency with which articles laudatory of massage appear in various periodicals. In Germany[20] the military authorities now require a semi-annual report from their surgeons upon the results of massage in injuries of joints, and the statistics of Gassner, Starke, Korner, and others clearly show the rapid results of this method, and the economy of time to the soldier. According to C. Krafft[21] (Lausanne), massage should be applied under aseptic conditions, and as soon as possible after an accident, in cases of contusion or sprain. He first washes the affected region with soap, and then shaves it. As lubricant he employs glycerine, to which mercury perchloride has been added, 1 in 1,000. Reclus'[22] treatment of sprain consists in the use of water at a temperature of 55° C., massage, and the application of an elastic bandage. Unless the lesion is exceptionally severe, recovery, he says, will be obtained by this means within a fortnight.

**Lower Extremity.**—Geo. J. W. Fowler[23] describes a remarkable instance of dislocation of the femur on to the pubes, with fracture of the neck of the bone. He figures the head and neck of the bone which he had successfully removed. At the recent annual meeting of the British Medical Association Sir William Stokes[24] opened a discussion on the diagnosis and treatment of the upper third of the femur, including the neck. His exhaustive paper was accompanied by many illustrations and valuable specimens, while his tabulation of the points of differential diagnosis of injuries in this region are well worth perusal. He formulated the following conclusions: (1) The alleged senile alteration in the angle between the axis of the cervix and shaft of the femur does not occur, and cannot therefore be regarded, as it has hitherto been, as a predisposing cause of fracture of the cervix femoris; (2) that the process of absorption in the case of fracture is probably due primarily to senile osteoprosis, and secondarily to the twofold action of the absorbents on the one hand and friction of the broken fragments on the other, and in the case of contusion, of impaired nutrition consequent on a low form of synovio-periosteal inflammation; (3) that in classifying fractures of the cervix or its base, a distinction should be made between those with and without penetration, and those with and without impaction; (4) that osseous union may take place in four out of the five different forms of fracture of the cervix; (5) that the further the fracture is from the base of the neck the less is the probability of osseous union; (6) that fracture of the cervix may occur previously to epiphysial junction; (7) that the differentiation of these injuries lies between fracture of the cervix or its base, luxation fracture of the acetabular brim, of acetabular fundus, and contusion; (8) that the principles of treatment should be fixation, rest, and moderate extension. Southam[25] records three cases which have been treated in the Manchester Royal Infirmary since he first drew attention to his method of treating impacted fractures of the neck of the femur by breaking down the impaction under anæsthesia. The results were satisfactory, especially as regards the correction of the eversion. As the patients were aged respectively 50, 65, and 75 years, they illustrate the fact that advanced age is in itself apparently no bar to this procedure, the fracture in each instance readily uniting with an abundant formation of callus after the impaction had been broken down.

[1] The Quarterly Med. Journal, January, 1896, p. 107. [2] Am. Jour. of the Med. Sciences, Nov., 1895; and Cent. für Chir., 1895, No. 22. [3] Am. Med. Surgical Bulletin, Oct. 1, 1895, p. 1,182; and Cent. f. Bakt. w. Parasith, 1895, xviii. vol. 2—3. [4] Beilage zum. Cent. für. Chir., 1895, No. 27; and Am. Jour. of the Med. Sciences, Nov., 1895 [5] The Medical Week, 1895, p. 530; Ibid. Nov. 22, 1895, p. 554. [6] The Medical Week, Nov. 1895. [7] The Quarterly Med. Journal, Oct. 1895. [8] N.Y. Med. Jour., Dec. 7, 1895. [9] Ep.B. M. Jour., Dec. 28, 1895; and Progrès Méd., Oct. 26. [10] N.Y. Med. Jour., Dec. 7, 1895. [11] Epit. B.M. Journ., Nov. 23, 1895; and Boston Med. and Surg. Journal, Sept. 19. [12] Wien. Klin. Rundschan, Oct. 20, 1895; and Ep. B.M.J., Nov. 23, 1895. [13] The Medical Week, Nov. 1, 1895, p. 519. [14] Lancet, Nov. 2, 1895, p. 1,094. [15] Am. Journ. of the Med. Sciences, Nov., 1895; and Beilage zum Centrelblat für Chir., 1895, No. 27. [16] Edinburgh Med. Jour., Oct. 1895, p. 351. [17] Epit. Brit. Med. Jour., Dec. 14, 1895, p. 94; and Méd. Moderne, Nov. 2, 1895. [18] Lancet, Nov. 16, 1895, p. 1,228. [19] The Hospital, Oct. 26, 1895, p. 61. [20] Therapeutic Gazette, Oct. 18, 1895, p. 764. [21] The Medical Week, Dec. 13, 1895. [22] The Medical Week, Nov. 22, 1895. [23] Brit. Med. Jour., Nov. 2, 1895, p. 1,101. [24] Brit. Med. Jour., Oct. 12, 1895, p 881. [25] Lancet, Dec. 21, 1895.

